# On Some Forgotten Formulas of L. de Broglie and the Nature of Thermal Time

**DOI:** 10.3390/e26080692

**Published:** 2024-08-16

**Authors:** Leonardo Chiatti

**Affiliations:** ASL Medical Physics Laboratory, Via Enrico Fermi 15, 01100 Viterbo, Italy; leonardo.chiatti@asl.vt.it

**Keywords:** thermomechanics, thermal time, wick rotation, Compton scale, quantum of action

## Abstract

From 1948 until around 1965, Louis de Broglie, awarded the Nobel Prize for Physics in 1929 for his fundamental contributions to quantum theory, pursued a systematic study of the formal analogies between wave mechanics and the thermomechanics of Boltzmann and Helmholtz. As part of this line of research, he produced several interesting observations, which were, however, published only in French, and, therefore, had a very limited diffusion. Here, we reconsider, in particular, a result of his relating to the analogy between the internal clock (de Broglie phase) of a free particle and a cyclic isothermal process in a thermomechanical system. We show that the fundamental equivalence obtained by him can be derived under more convenient hypotheses than the original ones, essentially tied to the quantization of the action exchanged by the particle with a suitable thermostat. In this emended formulation, the relations proposed by de Broglie describe the emergence of the particle proper time from a thermal background. They also suggest a specific physical meaning of the Wick rotation, often used in quantum mechanical calculations, and the thermal time that appears in it.

## 1. Introduction

In the 19th century, the search for a unified theory of mechanical and thermal phenomena mainly followed two very different directions. The first direction, which had its most eminent exponents in Maxwell and Boltzmann [1,2], was the statistical one; it aimed to express thermal and thermodynamical quantities, such as the heat exchanged between two systems, temperature and entropy, in terms of the statistical mechanics of the microscopic (disordered) degrees of freedom of the systems studied. The relationship between these quantities and macroscopic mechanical quantities, such as the kinetic energy or action, is, therefore, mediated by statistics.

A different approach, the thermomechanical one, instead, aimed to establish *direct*dynamical relationships between macroscopic mechanical quantities (such as action) and thermal quantities (such as entropy), without introducing statistical concepts. The major exponents of this line of thought were Boltzmann himself and Helmholtz [2,3]. This line of research identified, in fact, valid relationships between these two classes of quantities, but only for specific types of systems, such as closed cyclic systems. Due to its narrow field of applicability, this approach gradually lost ground compared to the other, and was eventually abandoned. Today’s statistical thermodynamics represents the mature development of the kineticstatistical approach, although the thermomechanical program is still the subject of active research, even with impressive proposals, e.g., [4,5,6,7,8].

The name of Louis Victor de Broglie (1892–1987) is commonly associated with his intuition of wave–particle dualism, described in his famous doctoral thesis [9], which represented a milestone in the development of quantum theory and earned him the Nobel Prize in 1929. Much less known are his efforts in the field of thermodynamics [10,11,12,13,14], to which he contributed with a careful study of a topic that was controversial for a long time: that of the relativistic extension of the theory. In fact, only starting from the 1970s did an acceptable agreement develop within the scientific community on a basic but not trivial question, that of the relativistic transformation of thermal and thermodynamic quantities. De Broglie’s contributions to this debate helped greatly to clarify the subtleties involved in the arguments.

De Broglie’s interest in thermodynamics, however, went beyond, towards a reconsideration of the ancient thermomechanical works of Boltzmann and Helmoltz on which he held regular cycles of lectures, whose materials are of exemplary clarity [10]. It is in this context that he came into contact with a result, obtained by Boltzmann in 1897 [15] and then forgotten, which particularly struck him. This is a relationship valid for systems with monoperiodic molecular motion, whose trajectories are subjected to a virtual variation between fixed extremes, separated by a time interval that is a multiple of the internal period *τ*. The variation of the trajectory involves both a variation *δQ* of the heat *Q* exchanged by the system along the natural trajectory in a period, and a variation *δA* of the mechanical (Maupertuis) action *A*, relative to the same period. The relationship is as follows:

*δQ *=* ν δ A* (1)

where *ν* = 1/*τ* is the frequency of the monoperiodic motion. If the heat *Q* is exchanged reversibly at a constant absolute temperature *T*, Equation (1) takes the form:

*TδS *=* ν δ A* (2)

where *S* is the exchanged entropy. From the relativistic point of view, *T* and *ν* transform as the reciprocal of the fourth component of a four-vector, while *S* and *A* are invariant. Therefore, (2) can be broken into two distinct relations [16,17,18,19,20,21,22]:

*kT *=* hν* (3)

*δS*/*k *=* δA*/*h* (4)

where *k* and *h* are constants having the dimensions of an entropy and an action, respectively. If they are, respectively, identified with the Boltzmann constant and the Planck constant, an interesting consequence is obtained. Let us consider a particle of mass *m* described by a plane wave function. In the rest frame, the form of this function is exp(2*πiνt*), where *t* is the particle proper time and *ν* = *mc*^2^/*h*. If we assimilate the particle to a monoperiodic system and apply (3) and (4) to it, we obtain:

*kT* = *mc*^2^ (5)

Therefore, the particle, even when it appears isolated in a vacuum, is in contact with a “hidden thermostat” with which it exchanges heat at the temperature *T* defined by (5). The variations of entropy and action are connected through Equation (4). In particular, an action variation of *h* (i.e., a quantum of action) corresponds to an entropy variation of *k*. Naturally, a limitation of the application of (3) and (4) to the de Broglie oscillation exp(2*πiνt*), the *internal clock* of the particle, is their derivation by variations of classical trajectories, which do not exist in the quantum domain. If, however, we gloss over this unpleasant aspect, we easily realize what is at stake: Equation (4) would have the potential to relate, at least in the case of a single particle, the principle of minimum action with that of maximum entropy, that is, the mechanics with thermodynamics; a connection mediated by wave–particle duality! This connection falls fully within the objectives of the Boltzmann–Helmholtz thermomechanical approach and represents a natural extension of the way in which de Broglie discovered wave–particle duality. We remember, in fact, that he achieved this important result [9] by equating the Fermat principle with the Maupertuis principle of least action, which required equalizing the velocity of the particle to the group velocity of the wave packet associated with it. This identification linked mechanics with optics. The present reasoning appeared, in de Broglie’s eyes, as a further connection between these areas of physics and thermodynamics.

However, de Broglie failed, by his own admission [22], to develop this connection into a complete form that satisfied him. The reflections reported in this section, and others that are not mentioned here, were published in internal notes and volumes as a corollary to the series of lessons and exclusively in French [16,17,18,19,20,21,22,23]. This is an element that has not favored their diffusion.

In the Materials and Methods section, we propose a different construction of relations (3)–(5), which makes no reference to classical notions of trajectory. We will show that these relations are a direct consequence of the application of elementary concepts of equilibrium thermodynamics to the notion of the renormalized particle. Starting from this basis, in the Results section, we give a simple physical interpretation of the Wick rotation, a mathematical operation often used in quantum mechanical calculations but normally considered as a simple computational trick. At the same time, a meaning is given to “thermal time”, often used in combination with this operation. In the Discussion section, the emergence of the particle proper time from a thermal background is contrasted with the open problem of the emergence of time as a spacetime coordinate in quantum gravity. The Conclusions section is a brief summary of the theses supported. The emergence of the de Broglie phase factor from heat exchange is illustrated, slightly more formally, in Appendix A.

Let us close this Introduction with a clarification: a significant part of de Broglie’s work on the “hidden thermodynamics of the isolated particle” concerns the introduction of an entropy function connected with the variance of the particle rest mass; this topic is intentionally excluded from the considerations developed in this work because, in our opinion, this function is distinct from the one that appears in formulas (2) and (4). The reasons for the simultaneous presence of two distinct entropy measures in quantum mechanics have been elucidated in a recent work [24]. The reader interested in this second entropy function can refer, in addition to the aforementioned works by de Broglie, also to the review work by Fronteau [25].

## 2. Materials and Methods

Real free particles are described by a wave function that conforms to the ordinary relativistic dispersion law:

*E*^2^ = *p*^2^*c*^2^ + *m*^2^*c*^4^ (6)

where *m* is the mass of the particle, *p* is the magnitude of its momentum, and *E* is its energy (*c* is the limit speed). These quantities are connected, respectively, to the wavelength *λ* and the frequency *ν* of the wave by the well-known relations:

*E* = *hν*  (Planck) (7)

*p* = *h*/*λ*   (de Broglie) (8)

where *h* is Planck constant or quantum of action. If the energy and momentum (vector) quantities are exactly definite, the wave is plane. Equation (6) admits a frame reference of rest in which *p* = 0, and, in this reference, the plane wave takes on the form already seen, exp(2*πiνt*), with *ν* = *mc*^2^/*h*. 

An important aspect of this “first quantization” description is that it is approximate, and ceases to be valid on the Compton scale [26], i.e., on time intervals smaller than *τ* = 1/*ν*, or on spatial intervals smaller than *cτ*. On this scale, the concept of propagation of the particle wave function is no longer applicable, because in fact there is no longer an entity to which this concept can be applied. The particle is dissolved into virtual particle–antiparticle pairs and the members of each pair interact with each other and with the members of other pairs through appropriate fields. The appropriate description at this level is that constituted by the second quantization, typical of quantum field theories. At the Compton scale relevant to a given mass level *m*, a dynamical equilibrium occurs between dissociations and recombinations of particle–antiparticle pairs of that mass. It is above this scale that spacetime appears “empty”, that is, devoid of particles of that mass. Real particles move within this “vacuum” as charges and masses *renormalized* by interactions with the surrounding virtual particles [27].

On the basis of these premises, we can now formulate our starting assumption: the renormalized particles, and the function exp(2*πiνt*) associated with them, are *mechanical* systems, existing in a coarse-grain description, in contact with an invisible *thermostat* constituted by the sea of non-renormalized virtual particles living on sub-Compton scales. We can say, using the language of 19th century thermomechanics, that renormalized particles are the slowly varying degrees of freedom of a system that also includes a multitude of other rapidly varying degrees of freedom (connected to virtual dissociations and recombinations). These degrees of freedom release energy in a *disordered* form, i.e., heat, to the renormalized particle; there is no production of “macroscopic” work, i.e., of forces, because this would violate the principle of inertia. The renormalized particle, therefore, exchanges heat with its environment, and it is in thermal equilibrium with it. Otherwise, the energy of the isolated particle would not be constant. The thermal equilibrium condition implies that the heat exchange occurs at a defined temperature, and is reversible. If we indicate the exchange temperature with *T*_0_, from the energy–time uncertainty relationship on the Compton scale, we have:

(*kT*_0_)*τ* = *h* (9)

Observing that *τ* = 1/*ν*, *ν* = *mc*^2^/*h*, one obtains:

*kT*_0_ = *mc*^2^ (10)

which is the new version of (5).

If *Q* and Δ*S* are, respectively, the heat and entropy exchanged by the renormalized particle with the thermostat in one cycle of the wave function, i.e., in an interval *τ*, we have:

*T*_0_Δ*S* = *Q* = *mc*^2^ (11)

in which *m* is the renormalized value of the mass; remember that this is the physically observed value of this quantity. The increase Δ*A* of mechanical action *A* in the same cycle is:

Δ*A* = (*mc*^2^)*τ* = *h* (12)

From (11) and (12) follows:

Δ*S*/*k* = Δ*A*/*h* = 1 (13)

Posing the de Broglie phase as *φ* = 2*πνt*, it is possible to define a cyclic time as *x∙τ*, being that *x* = [*φ* Mod(2*π*)]/(2*π*). The heat exchanged in the interval *dx* is then *Qdx* = *mc*^2^*dx*, and, from the relation *T*_0_*dS* = *Qdx*, taking into account (10), we obtain *dx* = *dS*/*k*. On the other hand, the increase *dA* of action in the interval *dx* is *dA* = *mc*^2^*τdx*, a relation giving *dx* = *dA*/*h* by virtue of (12). Then:

*dS*/*k* = *dA*/*h* = *dx* (14)

Equation (14) represents the new version of (4). In it, increments rather than variations appear, and the connection with the de Broglie phase is made apparent by the appearance of *x*. The meaning of the cyclic time *x∙τ*, which de Broglie called the “internal clock” of the particle, then becomes transparent. In the interval *x*∈ [0,1), the renormalized particle exchanges heat with the thermostat continuously, until reaching a total entropic exchange Δ*S*. At this point, corresponding to the instant *x* = 1, the thermostat transfers to the particle a finite quantity of heat −*Q* = −*mc*^2^ opposite to that exchanged during the interval, in a quantized way. The result is an entropic variation −Δ*S* which brings the renormalized particle back to the initial state *x* = 0. From here derives the cyclicity of time *x∙τ*, and, therefore, the cyclical nature of the internal clock.

The passage from the instant *x* = 1 to the instant, coinciding with it, *x* = 0 does not involve any increase in action because *dx* = 0; but it involves a finite discontinuous increment of entropy, which is brought back to the initial value. Ultimately, the heat exchanged in the interval *x*∈ [0,1] is zero, because the exchange in the interval [0,1) is compensated by the opposite exchange at *x* = 1. Considering that the exchange of work is also null, we obtain from the first law of thermodynamics a null variation in the internal energy of the renormalized particle. This result conforms to the cyclic nature of the transformation, because the internal energy is a state function.

However, it must be noted that, while the entropy variation is reset, the action *A* is instead added over the succession of cycle intervals. The action *A_n_* corresponding to *n* intervals is *A_n_* = *n*Δ*A* = *nh*, as a consequence of (13). Since *n* = *t*/*τ*, we have *A_n_* = *th*/*τ*. The particle proper time, *t*, which is the variable used by a macroscopic observer to coordinate the events of the particle history in its rest reference, is, therefore, defined as a non-cyclic variable as a consequence of the cumulative nature of *A_n_*. 

The proposed scheme can also be seen in the following terms. There are three systems affected by the transformations described, which are the thermostat, the virtual processes involved with the propagation of the particle, and the renormalized particle. The thermostat is a heat reservoir, while the renormalized particle is an action accumulator. The virtual processes act as an intermediary between the thermostat and the renormalized particle. They absorb heat from the thermostat undergoing a corresponding increase in action according to Equation (14). When the action increment reaches the critical value *h*, it is transferred to the renormalized particle, while the heat absorbed by the thermostat is returned to the latter at the same temperature. Consequently, the thermostat and the virtual processes are reset to the initial condition and the cycle starts again, while the action of the renormalized particle increases by an amount *h*. The cyclic transformation thus constructed is the basis of the de Broglie oscillation. The total action accumulated by the renormalized particle is *nh* with *n* integer, and it corresponds to the propagation of the particle over a *continuous* time interval of extension *nτ*. The origin of this interval is, therefore, thermal.

In this article we consider the situation in the rest frame of reference of the particle. In an inertial reference system that moves with speed *β* (in units *c*) with respect to the rest reference of the particle, the period *τ* is transformed according to the relation:

*τ* = *h*/*mc*^2^→*h*/[*mc*^2^/(1 − *β*^2^)^1/2^] = (*h*/*mc*^2^)(1 − *β*^2^)^1/2^ = *τ*_0_(1 − *β*^2^)^1/2^ (15)

where *τ*_0_ = *h*/*mc*^2^. The variable *x* is invariant as a consequence of (14). The internal time *x* then becomes *xτ’*/*τ*_0_, under the condition that (*xτ’*/*τ*_0_)[*τ*_0_(1 − *β*^2^)^1/2^] is equal to *xτ*_0_. This implies *τ’* = *τ*_0_/(1 − *β*^2^)^1/2^.

Recall that *mc*^2^ is the heat *Q*_0_ exchanged in one cycle of the de Broglie oscillation, measured in the rest frame of reference of the particle. Therefore, from relativistic transformations *T* = *T*_0_(1 − *β*^2^)^1/2^ and *Q* = *Q*_0_(1 − *β*^2^)^1/2^ of temperature *T* and heat *Q* [11,12,28,29,30], we obtain the following immediate generalization of (10):

*kT* = *Q* = *mc*^2^(1 − *β*^2^)^1/2^ (16)

It should be noted that this result is opposite to what one would intuitively expect, because the rest energy is divided by the Lorentz contraction factor and not multiplied by it. It can also be noted that, for *β*→ 1, the exchange temperature tends to absolute zero. 

The oscillation frequency *ν* = 1/*τ* is transformed as 1/[*τ*_0_(1 − *β*^2^)^1/2^] = ν_0_/(1 − *β*^2^)^1/2^, where ν_0_ = 1/τ_0_. Consequently, the phase increment relative to a period is the same, both in the rest reference of the particle (2*πν*_0_*τ*_0_ = 2*π*) and in the reference in which the particle is in motion (2*πντ* = 2*π*). Let us remember that it is precisely the need to satisfy this condition that leads to a contraction of the oscillation period according to (15), unlike the general law of dilation of time intervals in moving clocks [9].

The degenerate case *m* = 0 (for example, the photon) can be illustrated in the following terms. From Equations (6)–(8), we obtain, in the limit *m*→ 0, the relation *λν* = *c* typical of a propagation at the limit speed *c*. Consequently, there is no rest frame of reference and the period *τ* is expressed by Equation (15) in the double limit *m*→ 0, *β*→ 1; it coincides with the indeterminate form 0/0, and can, therefore, take on any positive real value. The same conclusion, therefore, holds for *ν* = 1/*τ* and for *λ* = *c*/*ν*. Equation (16) gives, in the same limit, an exchange temperature *T* = 0 and a total heat exchanged in the cycle *Q* = 0. The entropy associated with the cycle is, therefore, a zero-point entropy, whose value we assume as *k*. From Equation (14), finite values of *x* and *A* are obtained. The time variable marked by the particle’s clock is now *x*·*τ*, and it depends on the frame of reference, because *τ* is a function of the reference. The action associated with a cycle remains equal to *h*.

## 3. Results

The model proposed in the previous section links the cyclicity of the internal clock to the conservation of the rest energy which, according to (11), is the heat exchanged in each cycle. This heat is reversibly returned at the end of the cycle. So far, this construction, although it may be suggestive, constitutes a simple paraphrase of the de Broglie oscillation. To verify the actual relevance of the model, we need to see what its predictions are in a situation in which the de Broglie oscillation is removed. This means that the rest energy is no longer a constant of motion, connected to the other constants of motion *E* and *p* by the dispersion relation (6). This situation is typical of *virtual particles*. Virtual particles appear as fluctuations of quantum fields within the limits assigned by the uncertainty principle. If the hidden thermostat hypothesis is correct, these particles must correspond—in this description—to entropy fluctuations Δ*S*< 0 whose probability is defined by equilibrium statistical mechanics as [31]:

*P* = exp(Δ*S*/*k*) (17)

The instantaneous relations (14) do not depend on the actual existence of a cycle. Assuming their validity, we, therefore, have (Δ*A* > 0):

*P* = exp(−Δ*A*/*h*) (18)

Let us indicate with *d* the duration of the fluctuation in a reference in which it is only in energy and not in impulse, if such a frame of reference actually exists. In this case, writing the energy amplitude of the fluctuation as *mc*^2^, we have Δ*A* = *mc*^2^*d*. Therefore:

*P* = exp(−*mc*^2^*d*/*h*) (19)

At this point, it is possible to make two observations. The first is that the exponent of (19) is the same as that which would be obtained by acting on the de Broglie phase factor exp(2*πimc*^2^*t*/*h*) with the “Wick rotation” [32,33]:

2*πt*→*id* (20)

This observation explains the physical meaning of the operation, which is to remove the effects of the conservation principles (in the specific case, the conservation of rest energy) [24].

The second observation is that by defining a temperature *T* according to the condition:

*d* = *h*/*kT* (21)

Equation (19) is converted into a Boltzmann factor having the same numerical probability value:

*P* = exp(−*mc*^2^/*kT*) (22)

The meaning of the quantity *h*/*kT* is, therefore, to define the temperature *T* of a thermostat at which the probability of the energy fluctuation of amplitude *mc*^2^, given by (19), would equal the probability expressed by the Boltzmann factor (22). The quantity *h*/*kT* is often called “thermal time” (e.g., in [34]). In many cases, the substitution is used:

2*πt*→ *ih*/*kT* (23)

which combines (20) and (21) to transform quantum mechanics calculations into corresponding statistical mechanics problems [35].

Multiplying Equation (14) by *kT*_0_/*h* and taking into account Equation (10), we obtain, after integration:

*Q*(*x*)/*h* = *x*/*τ* (24)

where *Q*(*x*) is the heat exchanged in the interval [0, *x*) and the integration constant has been assumed to be zero. Equation (11) can be generalized by setting *Q*(*x*) = *kT*(*x*). Substituting into (24), we can then see that *x* is the ratio between the thermal time frequency *f_Th_* = *kT*(*x*)/*h* and the ordinary time frequency 1/*τ*. In other words, *x* = *τf_Th_* is the duration of the actual heat exchange *Q*(*x*), assumed as 1 the duration *τ* of the exchange of *Q*(1) = *mc*^2^. Tentatively, one can set *x* = (*q*_0_/*q*)^2^*mc*^2^ or *x* = (*q*/*q*_0_)^2^*mc*^2^ depending on whether the polarization of the vacuum shields the bare charge or strengthens it. Here, *q*(*x*) is the running charge and *q*_0_ = *q*(1) (evaluated in the rest frame of reference). The bare charge *q*(0) is infinite in the first case; it is instead zero in the second. The renormalized charge is connected to *q*_0_ (the running charge evaluated at the rest energy of the particle) by the renormalization semigroup equations. It can be noted that, setting *Q*(*x*) = *q*_0_^2^/*R*, we have, in both cases, *R* = *R*_0_/*x*∈ [*R*_0_, ∞) with *R*_0_ = *q*_0_^2^/*mc*^2^.

## 4. Discussion

The proposed scheme describes the emergence of the particle proper time from a thermal background. It is worth underlining that this topic is different from that, often discussed as a hypothesis in the quantum theory of gravity, of the emergence of time *as a coordinate of the spacetime continuum*, starting from a timeless, geometric or thermal background [36,37,38,39,40]. The first substantial difference is related to scale. The emergence of the proper time of a renormalized particle occurs on the Compton scale of the particle, for example around 10^−11^ cm for the electron. The emergence of time as a spacetime coordinate in quantum gravity would instead manifest itself on the Planck length scale ≈ 10^−33^ cm. We are, therefore, talking about two processes separated in scale by over twenty orders of magnitude.

A second important difference is that, obviously, the emergence of proper time concerns the single particle. The emergence of time as a spacetime coordinate, on the other hand, has consequences for all quantum fields and all their particles and is, therefore, of a more general scope.

Coming to more conceptual differences, the emergence of time discussed here is connected to the renormalization of charges and masses in quantum field theories, which gives rise to the description of first quantization as a low-energy approximation [27]. The topic discussed in quantum gravity instead concerns the unfreezing of the degrees of freedom associated with the spacetime metric, which is believed to occur on energies much lower than Planck’s, as a consequence of factors intrinsic to the specific quantum gravity theory adopted [36,37,38,39,40]. The low energy limit is constituted, in this case, by “classical” general relativity, and it forms the backdrop to the emergence of time in the sense discussed in this article. The latter occurs at energies (the rest energies of the elementary particles) negligible compared to that of Planck; thus, the geometric background can be assumed to be fully Minkowskian. In our analysis, we have implicitly assumed a fixed, dynamically inert background of this type.

The fact that the renormalized particle reabsorbs the emitted virtual particles makes it a sort of “thermodynamical black hole”. It is possible to apply to it the well-known Bekenstein relation on the maximum entropy *S* of a system of energy content *E* enclosed in a radius *r* [41]:

*S*/*k *=* 4πrE*/*ħc* (25)

Posing *E*= *Q*(*x*) = *mc*^2^*x*, *r* = *ħ*/4*πmc*~*h*/*mc*, one obtains *S*/*k* = *x*, that is, Equation (14). In other words, the entropy exchanged by the particle during the de Broglie oscillation is the Bekenstein maximum entropy and the Compton scale is the radius of the horizon associated with this exchange.

## 5. Conclusions

In this short note, the relations (3)–(5) proposed by de Broglie in his research program on the “thermodynamics of the isolated particle” have been reconsidered. After having very briefly illustrated the main steps in the derivation of these formulas, the major drawback of this derivation has been highlighted. It starts, in fact, from a result of Boltzmann thermomechanics for monoperiodic systems, Equation (1), which is related to variation operations on the classical trajectories of such systems. This concept is foreign to quantum theory. It is possible to derive the analogous expressions (11)–(14), however, with increments in place of variations. This difference makes any obvious “thermomechanical” connection between the principle of minimum action and the condition of maximum entropy evaporate, but it rests, in our opinion, on a clearer vision of what the “thermodynamics of the isolated particle” could be. The hidden thermostat becomes the set of sub-Comptonian processes to which the renormalized particle, physically detectable as an asymptotic state, is coupled. 

This thermostat exchanges heat with the renormalized particle in two ways, one continuous, the other discontinuous, generating an internal cyclic time of the particle which is nothing other than the de Broglie phase. While the total entropic exchange is reset to zero at each cycle, the mechanical action (connected to entropy) is cumulated over subsequent cycles, leading to the emergence of the proper time of the particle. The duration of the particle in its proper time is synonymous with the cyclic heat exchange with this thermostat. According to the proposed reading, therefore, the particle proper time (and, therefore, its rest frame of reference) emerges from a thermal background. This emergence is different from that of time as a spacetime coordinate, which according to different models of quantum gravity, would manifest itself on the Planck scale.

The proposed model can also be applied, at least within certain limits, to virtual particles. This leads to the explanation of the physical meaning of the Wick rotation and thermal time, two (often associated) concepts frequently used in quantum mechanical calculations, but normally considered as simple formal tools. The effect of the Wick rotation is to remove from the dynamics the conservation principles (specifically, the conservation of the rest energy of the particle) that are closely associated with the quantum phase, transforming real particles into virtual particles or fluctuations in a thermal bath. The meaning of thermal time is instead to define the temperature of the thermostat at which the probability of a virtual process equals the Boltzmann order factor.

## Data Availability

No new data were created in this study.

## Appendix A

Let us consider, for every value of *x* = *Q*(*x*)/*mc*^2^∈ [0,1], the following geometric locus on the complex plane:

*A*(*x*) = {*x*·exp[2*πiS*(*y*)/*k*] || 0 ≤ *y* ≤ *x*} (A1)

*A*(*x*) evolves in *x*, starting from the origin of the plane, as an arc with radius *x* and opening 2*πS*(*x*)/*k*. For *x* = 1, this arc closes in a circle, which immediately collapses into the origin, and, from there, a new cycle starts again. Let us consider the sequence of circles *x* = 1:

*A_n_*(*x*) = {exp[2*πiS*(*y*)/*k*] || 0 ≤ *y* ≤ 1} (A2)

where *n* is the cycle repetition index. For Equation (14), we have:

exp[2*πiS*(*y*)/*k*] = exp[2*πiA*(*y*)/*h*] = exp[2*πimc*^2^*t_P_*/*h*] (A3)

with *t_P_* ∈ [0, *τ*]. Connecting the variable *t_P_* on the various subsequent cycles:

*nτ* + *t_P_*→*t_P_* ∈ (−∞, +∞) (A4)

*t_P_* becomes the proper time of the renormalized particle, defined by the sequence {*A_n_*(1) || *n*∈*Z*}. We note that the connection (A4) is only possible for *x* = 1; for 0 ≤ *x* < 1, the single arc does not close and arcs relating to subsequent cycles cannot, therefore, be connected at the ends in a continuous way. Equation (A3), under condition Equation (A4), becomes the de Broglie oscillation of the renormalized particle. The latter exists for *x* = 1. Equation (A3) expresses, according to the Born rule, the equiprobability of all points in the *t_P_* domain, i.e., the conservation of the renormalized particle in this domain.

The radius *x* of the arc (A1) is a measure of the exchanged entropy, while the angular opening of the arc measures the accumulated action. The single cycle describes the conversion of entropy *k* into action *h*.
